# Regime Shift in an Exploited Fish Community Related to Natural Climate Oscillations

**DOI:** 10.1371/journal.pone.0129883

**Published:** 2015-07-01

**Authors:** Arnaud Auber, Morgane Travers-Trolet, Maria Ching Villanueva, Bruno Ernande

**Affiliations:** 1 IFREMER, Laboratoire Ressources Halieutiques, 62321, Boulogne-sur-Mer, France; 2 IFREMER, Unité Sciences et Technologies, 29280, Plouzané, France; Aristotle University of Thessaloniki, GREECE

## Abstract

Identifying the various drivers of marine ecosystem regime shifts and disentangling their respective influence are critical tasks for understanding biodiversity dynamics and properly managing exploited living resources such as marine fish communities. Unfortunately, the mechanisms and forcing factors underlying regime shifts in marine fish communities are still largely unknown although climate forcing and anthropogenic pressures such as fishing have been suggested as key determinants. Based on a 24-year-long time-series of scientific surveys monitoring 55 fish and cephalopods species, we report here a rapid and persistent structural change in the exploited fish community of the eastern English Channel from strong to moderate dominance of small-bodied forage fish species with low temperature preferendum that occurred in the mid-1990s. This shift was related to a concomitant warming of the North Atlantic Ocean as attested by a switch of the Atlantic Multidecadal Oscillation from a cold to a warm phase. Interestingly, observed changes in the fish community structure were opposite to those classically induced by exploitation as larger fish species of higher trophic level increased in abundance. Despite not playing a direct role in the regime shift, fishing still appeared as a forcing factor affecting community structure. Moreover, although related to climate, the regime shift may have been facilitated by strong historic exploitation that certainly primed the system by favoring the large dominance of small-bodied fish species that are particularly sensitive to climatic variations. These results emphasize that particular attention should be paid to multidecadal natural climate variability and its interactions with both fishing and climate warming when aiming at sustainable exploitation and ecosystem conservation.

## Introduction

Regime shifts are substantial, abrupt, persistent, often irreversible, reorganizations in the structure, functioning and feedbacks of an ecosystem or some of its components [1]. Unfortunately, our knowledge about the respective influence of various forcing factors and the mechanisms that generate regime shifts in marine ecosystems still remains limited [2] and impedes the development of proper management responses. This is especially true for exploited ecosystem compartments such as marine fish communities. Key drivers of regime shifts are abiotic and biotic processes as well as habitat restructuration [3]. More specifically, drivers of regime shifts in marine fish communities can include climate forcing [4] and anthropogenic pressures [5] as components that operate synergistically, which may render the quantification of their relative contributions difficult to achieve [3]. Regime shifts in marine communities that occur concurrently with climate-driven transitions in physical oceanographic conditions [2,6] may propagate through multiple trophic levels including fish [7] and on large geographical scales resulting in various and alternating quasi-stable states [1]. Evidence of regime shifts has been mounting over the last three decades, notably in several Northeast Atlantic marine systems where intense and rapid changes in phytoplankton [2,3], zooplankton [8,9] or fish compartments [10] were observed. Biological responses in fish ranged from changes in abundance (e.g., [2]) and distribution (e.g., [11]) of communities to changes in their structural properties [12,13]. However, most studies focusing on marine fish considered only a few species [2,14–16]. Among those that focused on entire fish communities, a majority used an integrative approach combining fish community data with those of several other biotic compartments [17]. It results that very few studies have explicitly investigated fish community structure dynamics. Moreover, these either focused on very restricted, most often coastal, areas and thus were not representative of the fish community at the scale of the entire marine ecosystem to which they belong [10,12], or did not investigate the potential drivers of community dynamics [13]. Therefore, the dynamics of marine fish communities and their drivers remain poorly documented and understood, especially at the scale of entire marine ecosystems and in the context of regime shifts.

Most studies on regime shifts in the North Atlantic focused on the influence of natural high-frequency (intradecadal) variations in atmospheric and oceanic circulation, measured by indices such as the North Atlantic Oscillation index (NAO) or the Gulf Stream North Wall index (GSNW), on biotic changes [6]. In the last decades, pervasive climate changes in the Northern Hemisphere raised questions on the effects of large multidecadal variations on marine ecosystems [18]. One low-frequency climate phenomenon that has received little attention so far is the Atlantic Multidecadal Oscillation (AMO), which is defined as a basin-wide multidecadal (>50 years) climate oscillation with alternating cool and warm phases representing global variations in sea surface temperature (SST) over the Northern Hemisphere [19]. Several studies showed SST warming from 0.2 to 0.6°C in the North Atlantic in the late 1980s [20,21], especially since the mid-1990s [21], and since the beginning of the 1990’s in the Bay of Biscay [22]. SST is recognized as one of the major factors controlling fish dynamics [11], thereby supporting the relevance of the AMO index for assessing the influence of SST natural multidecadal cycles on fish communities. To this day, in comparison with NAO, relatively few studies attempted to relate ecological changes to the AMO [23, 24]. In the North Atlantic, most studies are limited to the subartic regions and are usually validated by plankton and long-term catch data sets of commercial species [18]. Several studies related directly the AMO to abundance changes of North Atlantic salmon [25,26], Atlantic cod, herring [18] and plankton [24,27] and some linked it to habitat switching of two pelagic fishes in the North Atlantic [28]. In some cases, AMO effects on fish abundance declines were due to the species poleward migrations [11] aggravated by heavy fisheries or nutrient limitations [23, 26]. Recently, a special issue on AMO on marine ecosystems was published [29] featuring ecological responses to this index of fish communities/populations [18, 26, 28], diatom [27], zooplankton abundance [18, 27] and barnacle dynamics [30]. In the western English Channel, AMO events that broadly matches the Russell cycle [24,31] have been related to the large changes in abundance of several mesozooplankton species (chaetognaths, larvae of decapod crustaceans and pilchard eggs) [22], barnacles [30] and pelagic fishes [28,30]. The Russell cycle, the evidence of which goes back to the mid-19^th^ century, is considered as one of the earliest observed regime shifts [24,30,32].

In addition to climate forcing, changes in fish community dynamics are also induced by anthropogenic activities such as fishing [33]. Fishing is known as a strong forcing factor causing profound fish community reorganization and diversity changes in terms of taxonomic composition and/or relative abundances [33] as well as in terms of size spectrum [34]. The reaction of ecosystems to overexploitation goes beyond the decline of target species’ abundance leading to stock collapse in some cases [33–36], especially for populations of low demographic resilience [5,37]. Other indirect responses include the reorganization of community structure [38], changes in the size spectra, numbers, and diversity, and fishery-induced trophic cascades [36,38]. Combined with climate variability, such perturbation could therefore have a considerable impact on ecosystems [5]. Identifying natural and anthropogenic drivers of changes in marine communities and disentangling their influence are critical tasks for understanding biodiversity dynamics and properly managing living resources [39].

Based on 24 years of scientific surveys, we investigated temporal changes in the exploited fish community of the eastern English Channel (EEC) taken as a case study. We chose this area of interest since compared to adjacent ecosystems, the occurrence of regime shifts has not yet been investigated despite the availability of ecological and fisheries data time-series from annual scientific surveys and commercial fisheries monitoring. Here, we assess the relative effects of fishing and climate on the first documented regime shift in the EEC fish community structure and test for the relationship between multidecadal climate variations and this shift.

## Materials and Methods

We started by characterizing and testing for the existence of a regime shift in the EEC fish community by using multivariate (taxonomic structure) and univariate (diversity indices, mean body length, and temperature preferendum) descriptors. Then, we assessed whether the shift in community structure was related to climatic and/or fishing pressures. For climatic factors, we considered (i) basin-wide climatic indicators of various variability modes, i.e. multidecadal with the Atlantic Multidecadal Oscillation index (AMO) and intradecadal with the North Atlantic Oscillation index (NAO) and the Gulf Stream North Wall index (GSNW); and (ii) local physico-chemical parameters, i.e. sea surface temperature (SST), salinity (SSS) and dissolved oxygen concentration (SSO2) at sampling sites (see S1 Table). Fishing pressure was considered through 3 indices (F_*pelagic*_, F_*demersal*_, and F_*benthic*_) calculated each year as the 1-year lagged weighted average fishing mortality rates of pelagic, demersal and benthic fish species analytically assessed in the area, the averages being weighted by the landings of each species in the area.

### Fish community sampling

The fish community (including cephalopods) of the EEC (area VIId defined by the International Council for the Exploration of the Sea, ICES) was sampled during the Channel Ground Fish Survey (CGFS) operated on RV ‘Gwen Drez’, in October of each year since 1988. The sampling scheme was spatially stratified by subdividing the area into 15’×15’ rectangles (S1 Fig) in each of which at least one 30-min haul was carried out during daylight hours at an average speed of 3.5 knots. A high (~3 m) vertical opening bottom trawl (GOV) with 10 mm stretched mesh size in the codend was used. The stratified sampling scheme aimed to achieve 90 to 120 hauls depending on weather conditions. After each haul, all captured fishes and cephalopods were identified, weighed, and counted and a subset of species were measured (total length) to the nearest inferior centimeter. A total of 77 taxonomic groups was recorded over the study period. The abundance indices at each sampling station were provided by the ICES data portal [40] and were standardized to numbers of individuals per km^2^.

### Climatic factors

An overview of climatic and fishing pressure data used and their sources are given in S1 Table. Climatic conditions potentially affecting fish communities were described using ocean-wide climatic indices and local parameters. Regarding the former, the following indices were used: annual NAO, AMO and GSNW index. The NAO is an intradecadal basin-scale alternation of atmospheric mass over the North Atlantic between the high atmospheric pressures centered on the Azores and low atmospheric pressures around Iceland [41]. The index used here is based on the difference of normalized sea level atmospheric pressure between Lisbon, Portugal and Stykkisholmur/Reykjavik, Iceland since 1864 [41]. This oscillation has been correlated with a large range of physical (e.g. paths/intensity of storms, precipitations patterns, etc.) or biological indicators (e.g. zooplankton abundance, productivity) [42]. The annual NAO index for the period 1988–2011 was obtained from the National Oceanographic and Atmospheric Organization [43] (S1 Table).

The AMO refers to a 60–80 years cycle of the North Atlantic SST [24]. Its determinism is currently not fully understood nor is it predictable. The AMO index is computed as a monthly area-weighted average of SST anomalies over the North Atlantic (from 0 to 70°N). The obtained time series is then detrended in order to remove the effect of global warming. This index is correlated to air temperature, rainfall over the Northern hemisphere and positive (negative) phases are associated with warm (cool) and drought (wet) periods [44]. This index has been recently reported to influence plankton and fish abundance in some North Atlantic ecosystems [24,29]. AMO values were provided by the NOAA, US [45] (S1 Table).

The GSNW index is an indicator of the latitudinal position of the northern edge of the Gulf Stream that is marked by a sharp fall in temperature. The index is computed as the first principal component of the correlation matrix between time-series of latitudes of the north wall at six different longitudes (79, 75, 72, 70, 67, and 65°W). The Gulf Stream dynamics and variability in its path and flow have been reported to influence coastal fish [46], squid [47] and phytoplankton [48] populations, but also ecosystems in both the Northwest and Northeast Atlantic [49,50]. The GSNW index values were obtained from the Plymouth Marine Laboratory [51] (S1 Table).

Regarding local parameters, the following physico-chemical factors in the EEC were considered: sea surface temperature (SST), salinity (SSS) and dissolved oxygen (SSO2). No empirical data covering the whole EEC and time period was available. These parameters were thus extracted from output predictions of the NORWegian ECOlogical Model [52,53] (S1 Table). NORWECOM is a coupled 3D physical, bio-chemical model for the North Sea and the English Channel that provides monthly averages of various climatic parameters at a geographical resolution of 0.1 degree. More precisely, monthly averages of SST, SSS and SSO2 were extracted for the study time period at each cell of the model belonging to the fish community sampling area (S1 Fig). These data points were then averaged across spatial locations to obtain monthly means at the scale of the whole EEC that were used either directly −mean temperature, salinity, and dissolved oxygen concentration in October (SST_oct_, SSS_oct_, SSO2_oct_; S1 Table) as well as minimal and maximal temperatures, salinities, and dissolved oxygen concentrations (SST_min_, SST_max_, SSS_min_, SSS_max_, SSO2_min_, SSO2_max_; S1 Table) of each year− or after annual averaging −mean annual temperature, salinity, and dissolved oxygen concentration (SST_an_, SSS_an_, SSO2_an_; S1 Table). Only sea surface values were considered as the water column of the EEC is generally well mixed and rarely stratified. In any case, temporal trends in sea surface values were clearly indicative of temporal trends of the whole water column.

### Fishing pressure

Fishing pressure was accounted for by using three different fishing mortality indices, F_*pelagic*_, F_*demersal*_, and F_*benthic*_, for pelagic, demersal and benthic species, respectively. These were estimated each year as the 1-year lagged landing-weighted average fishing mortality rates available for stocks assessed by ICES working groups, namely mackerel (Working Group on Widely Distributed Stocks) and herring (Herring Assessment Working Group) for pelagic fish, cod and whiting for demersal fish (Working Group on the Assessment of Demersal Stocks in the North Sea and Skagerrak), and plaice and sole for benthic fish (Working Group on the Assessment of Demersal Stocks in the North Sea and Skagerrak). Each of the three fishing mortality indices corresponds to the average of the fishing mortalities of the two assessed stocks considered (either pelagic, demersal or benthic) weighted by the landings of each of these two stocks. Globally, the fishing mortality rates of these 6 stocks–the only ones analytically assessed in the EEC–were considered representative of the global fishing pressure on the fish community of the EEC, as these species accounted for more than 60% of the total landings in the area and are caught by mixed fisheries. The 1-year lagging accounted for the fact that annual instantaneous fishing mortality rates of a given year are expected to affect the abundance of target fish stocks the year after. We considered the pelagic, demersal and benthic compartment separately as these are targeted by different types of fishing gears. Fishing mortality rates of the different stocks as well as landing statistics were extracted from the ICES Stock Assessment Summary database and Catch Statistics database [54] (S1 Table).

### Analysis of community dynamics

Only taxa with abundance above 0.1% of the total abundance across the study period were included in this study [55], which resulted in a collection of 55 taxa of fish and cephalopods (S2 Table). All statistical analyses were performed using packages *vegan*, *mvpart*, *segmented*, *forecast* and *MVPARTwrap* under the R environment (R Core Team, 2012). Potential temporal autocorrelations of explained variables were checked before all statistical analyses and effects were declared statistically significant at the 5% level.

Spatially aggregated abundance indices at the scale of the whole EEC were used in the analyses described below unless otherwise specified. Aggregated indices were computed for each species and each year as the weighted mean of the numbers of individuals per km^2^ in CGFS rectangles using the surface of the rectangles as weight (notice that the surface of coastal rectangles may vary; see S1 Fig). Whenever several hauls occurred in the same rectangle, the number of individuals per km^2^ in the rectangle was estimated as the arithmetic mean across hauls. The resulting data was a matrix of spatially aggregated abundance indices per species (columns) and per year (lines) that represents the times series of the community taxonomic structure and to which we will refer to as the ‘species abundance matrix’.

Fish community dynamics were first analyzed by nonmetric multidimensional scaling (nmMDS) on the species abundance matrix using the Bray-Curtis distance [56]. Due to very low stress values, the first axis of the nmMDS (nmMDS1) was then used as an index of community structure for illustrative purposes. The existence and timing of a shift in community structure was assessed by chronological clustering using a multivariate regression tree (MRT) with the species abundance matrix as the explained matrix and time as the explanatory variable [56].

In addition to multivariate analyses, the time-series of three diversity indices were computed in each haul and analyzed: taxonomic richness (S), Pielou’s evenness (J) and Shannon diversity (H). Taxonomic richness (S) relates to the number of taxa, mostly at the species level but also at the genus level, whenever finer identification was impossible (for 8 taxa of 55). Evenness (J) refers to the balance of abundance distribution across species in the community. Shannon diversity (H) is a measure of taxonomic diversity that combines evenness and richness. The last two indices were computed as follow: *H* = −∑_*i*_ *p*_*i*_ log_2_(*p*_*i*_) and *J* = *H* / *H*_max_ with *H*_max_ = log_2_(*S*), where *p*_*i*_ is the relative abundance of the taxonomic group *i* and *H*_max_ is the maximum diversity that would be reached if all taxonomic groups were equally abundant. The temporal dynamics of these three indices were analyzed by piecewise linear regression (PWR) against time in order to test for both linear temporal trends and for the existence of breakpoints in their time series.

### Characterization of the shift in community structure

The shift in community structure was first characterized by testing for changes in the abundance of each species, between the period before and that after the shift. A Monte-Carlo permutation test was then performed on the spatially aggregated abundance indices using the ‘max statistic’ method described below in order to account for the increase in the family-wise type 1 error rate due to multiple testing [57]. Lines of the species abundance matrix corresponding to years were randomly permuted between the period before and that after the shift. This randomized any potential association between the abundance index of each species and the period while preserving any correlative structure between species abundance indices themselves. A one-way ANOVA with period as the explanatory variable was then performed on the abundance index of each species in the permuted matrix and the maximum F value across species was recorded. This procedure was repeated 5000 times and the resulting distribution of permuted maximum F values was used as the empirical null distribution against which observed F values, computed through one-way ANOVAs on the actual time series of species average abundance indices, were tested. A change in species abundance was then declared significant when less than 5% of the permuted maximum F values were larger than the observed F value.

The shift in community structure was also characterized in terms of changes in individual body size. Individual total body length data from the CGFS survey were available for 50 species of 55 (see S1 Table), and used for computing the annual abundance-weighted mean individual body length observed in the community. The resulting time-series was analyzed by piecewise linear regression against time in order to test for a shift in individual body size.

Finally, the most negatively and positively impacted species −defined respectively as the first and last decile of the distribution of absolute changes in abundance before and after the shift− were compared in terms of their thermal preferendum. The temperature preference of each species was assessed from observation data available on the Global Biodiversity Information Facility, a web data portal aiming to provide free and open access to global data on biodiversity [58]. Each species record is accompanied by a set of physico-chemical parameters that characterizes the observation site. We estimated the thermal preferendum of a species as the mean temperature across all its records. A one-way ANOVA was then performed for comparing the thermal preferendum of negatively and positively impacted species.

### Effects of climatic conditions and fishing pressure on community structure

A redundancy analysis (RDA) was used in combination with Monte-Carlo permutation tests to analyze the relationships between the species abundance matrix and a matrix comprised of climatic conditions −including ocean-wide climatic indicators, AMO, NAO, and GNSW together with local physico-chemical parameters, SST_oct_, SST_min_, SST_max_, SST_an_, SSS_oct_, SSS_min_, SSS_max_, SSS_an_, SSO2_oct_, SSO2_min_, SSO2_max_, and SSO2_an_− and fishing mortality indices, F_*pelagic*_, F_*demersal*_, and F_*benthic*_. The species abundance matrix was Hellinger-transformed to reduce the importance of large abundances and to alleviate the problem of double zeros [56]. This analysis was performed on a shorter time-series (1988–2008) because temperature, salinity and oxygen data were not available after 2008.

Collinearity between explanatory variables in the RDA model could bias the estimated regression coefficients, these being unstable and inconsistent [56]. Therefore, we examined possible linear dependencies between explanatory variables by computing the variables’ variance inflation factors (VIF) which measure the proportion by which the variance of a regression coefficient is inflated in the presence of other explanatory variables. VIFs should ideally be below 10, those above 20 indicating strong collinearity.

Several VIFs being larger than 20, the model was reduced in search for parsimony and in order to avoid collinearity problems. Stepwise selection of explanatory variables was performed on the basis of *p*-values obtained by Monte-Carlo permutation tests. All VIFs after model reduction were below 10. The amount of variance in the community structure time-series explained by the selected explanatory variables was evaluated by a variation partitioning analysis [56].

In a second step, a multivariate regression tree of the species abundance matrix against the selected variables was used in order to assess hierarchically the implication of explanatory variables in the shift in community structure.

## Results

**Fig 1:**
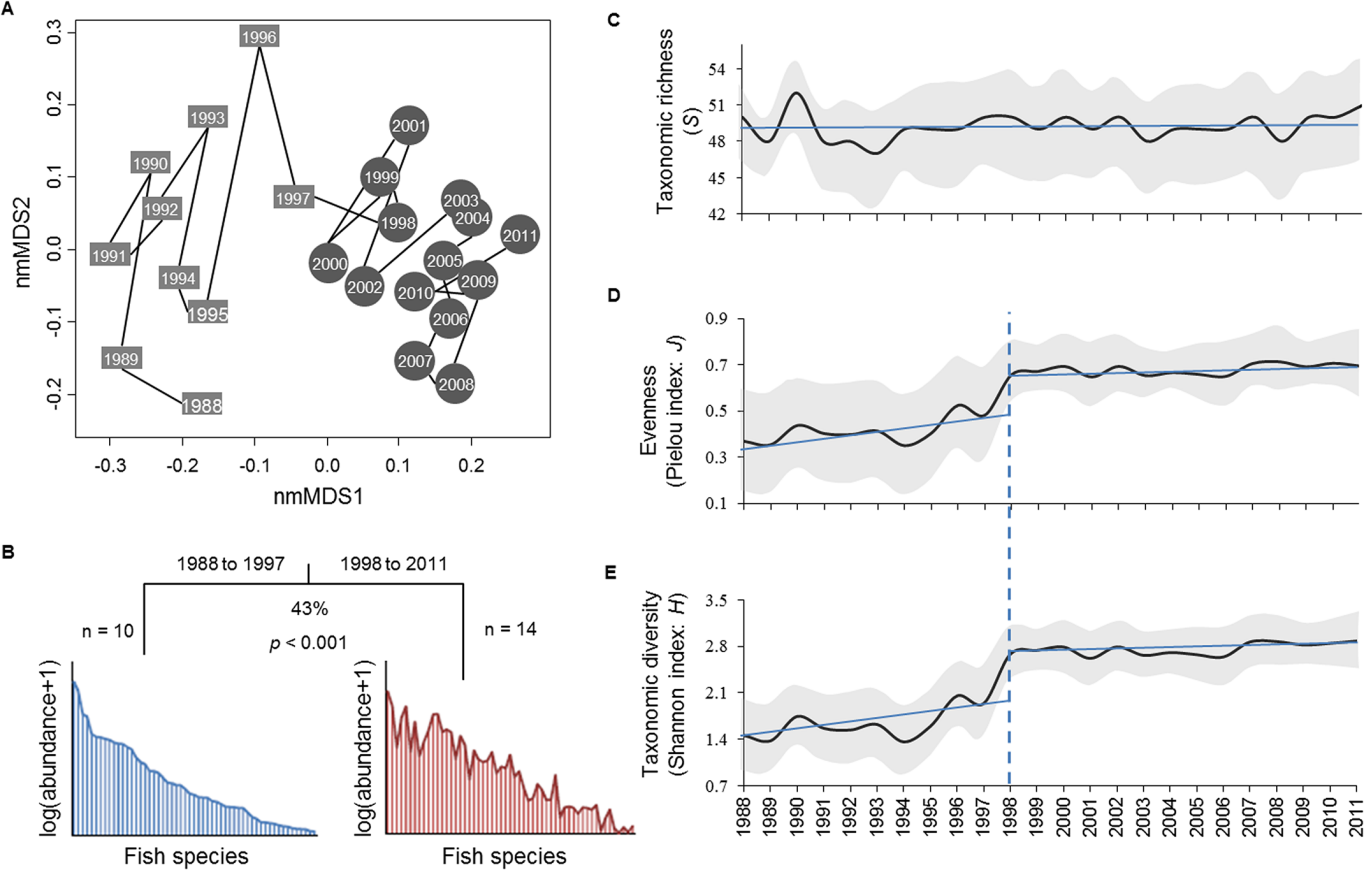
Temporal dynamics of fish community. **A**. nmMDS biplot illustrating temporal changes in the community structure. Squares and circles respectively depict the years before and after the regime shift, detected by MRT. Axis 1 appears particularly discriminant in this respect. **B**. Chronological clustering by MRT and the two successive structures of the fish community. **C**. Taxonomic richness. **D**. Pielou’s evenness. **E**. Shannon diversity. Black lines represent average indices and shaded areas are standard deviation intervals. Straight blue lines represent the PWR results on indices while dashed ones indicate breakpoints in the time series whenever detected.

Temporal dynamics of the fish community structure (55 species; S2 Table), described by non-metric multidimensional scaling (nmMDS), revealed an abrupt change from 1995 to 1998: taxonomic structure was relatively stable before 1995 but then changed rapidly before stabilizing again from 1998 onward (Fig 1A). Given the abruptness and persistence of the observed change, it could qualify as a regime shift. A multivariate regression tree (MRT) of community data against time detected the existence of a significant change in community structure between 1997 and 1998 (permutation test: *p* < 0.001; Fig 1B). This change represents 43% of community structure variation over the study period. Concurrent changes in diversity were examined by piecewise linear regression (PWR) of various indices on time. Taxonomic richness was stable through time (PWR: $F_{19}^{4}=0.48$, *p* = 0.751; Fig 1C and S3 Table) whereas evenness (Pielou) and consequently diversity (Shannon), almost doubled from 1994 to 1998, PWR detecting a significant breakpoint in 1998 for both of them (Pielou: $F_{19}^{4}=106.1$, *p* < 0.001, Fig 1D and 1E, S3 Table; Shannon: $F_{19}^{4}=103$, *p* < 0.001, Fig 1E and S3 Table).

**Fig 2:**
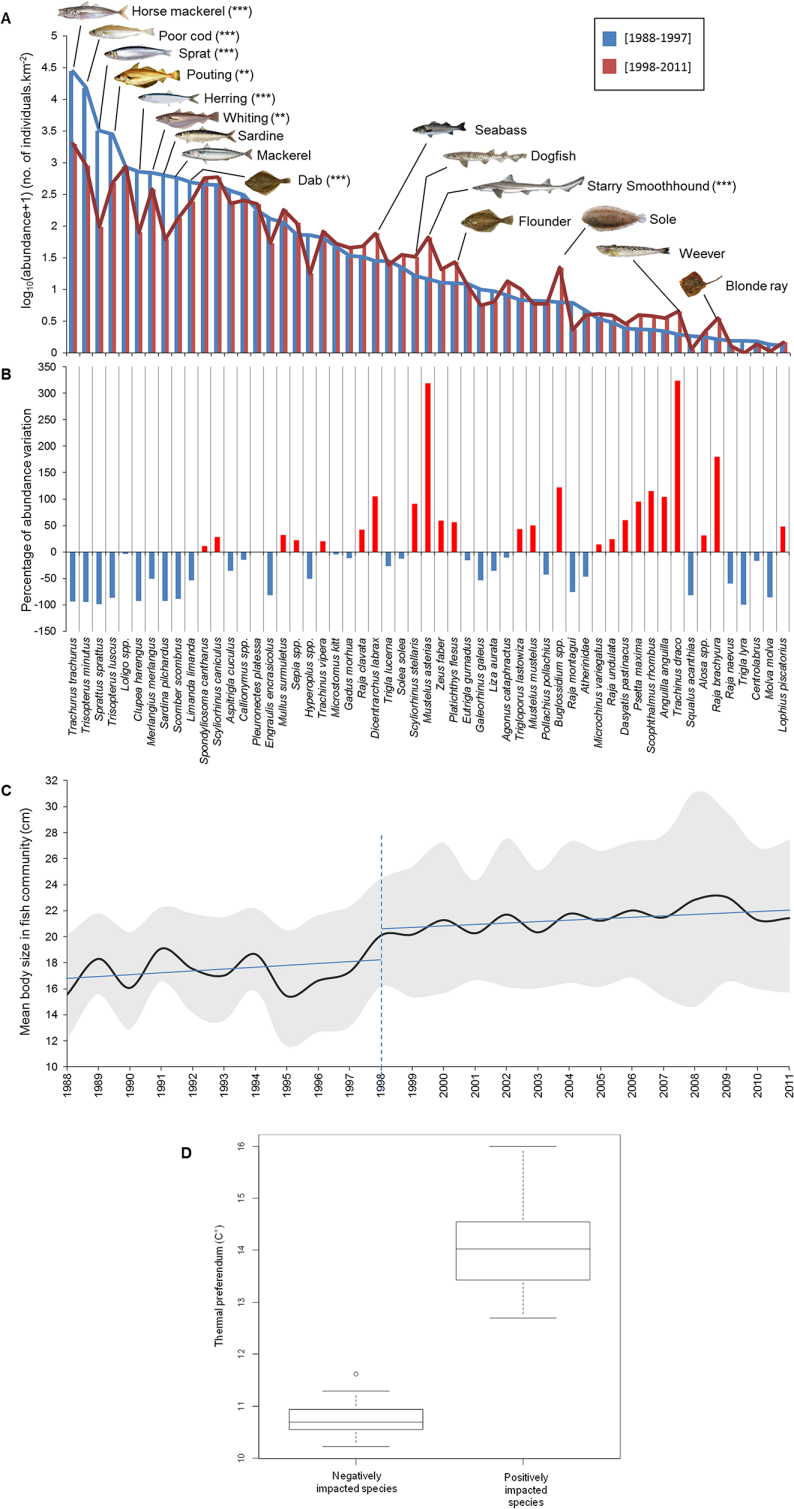
Changes in fish community structure. **A**. Taxonomic structure based on species abundance before and after the regime shift (species are ordered according to their mean abundance before the shift). The species pictured correspond to the first and last decile of the distribution of absolute changes in abundance before and after the shift. Results of permutation tests for change in each species abundance before and after 1998 accounting for multiple testing: ***: *p* < 0.001; **: 0.001< *p* <0.01; *: 0.01< *p* <0.05. **B**. Percent abundance variation between the two periods. For a given species *i*, the percentage is computed as follows: (N*i*_after 1997_ –N*i*_before 1997_) ×100/ N*i*_before 1997,_ with N*i* the average abundance of species *i*. **C**. Evolution of mean body length in fish community. Line types are as in Fig 1C to 1E. **D**. Boxplot representing thermal preferendum of negatively and positively impacted species.

The regime shift was mainly characterized by a decrease in small-bodied fish abundance while several subordinate larger species increased in abundance. The main negatively impacted small-bodied species (first decile of absolute change in abundance) were *Trachurus trachurus* (horse mackerel), *Trisopterus minutus* (poor cod), *Sprattus sprattus* (sprat), *Trisopterus luscus* (pouting), *Clupea harengus* (herring), *Merlangius merlangus* (whiting), *Sardina pilchardus* (sardine), *Scomber scombrus* (mackerel) and *Limanda limanda* (dab), whereas the most positively affected species (last decile) were *Dicentrarchus labrax* (seabass), *Scyliorhinus stellaris* (nursehound), *Mustelus asterias* (starry smoothhound), *Platichthys flesus* (flounder), *Buglossidium spp*. (solenette), *Trachinus draco* (greater weever) and *Raja brachyura* (blonde ray) (Fig 2A). The total abundance of the main declining species was reduced from 64777 to 4605 ind/km² (i.e., -93% of abundance variation, see Fig 2B) while the total abundance of increasing species raised from 146 to 307 ind.km² (+103% of abundance variation, see Fig 2B) from before to after the shift. Community size structure was affected with mean body length increasing from 17.2 ± 4.1 cm before 1998 to 21.4 ± 5.9 cm after 1998, with a significant breakpoint detected in 1998 (PWR: $F_{20}^{3}=34.67$, *p* < 0.001; Fig 2C; S4 Table). The thermal preferendum of negatively impacted species appeared significantly colder than that of positively impacted ones (ANOVA: $F_{48}^{1}=404.9$, *p* < 0.001; Fig 2D). Qualitatively similar results were obtained when considering species biomass instead of abundance (results not shown).

**Fig 3:**
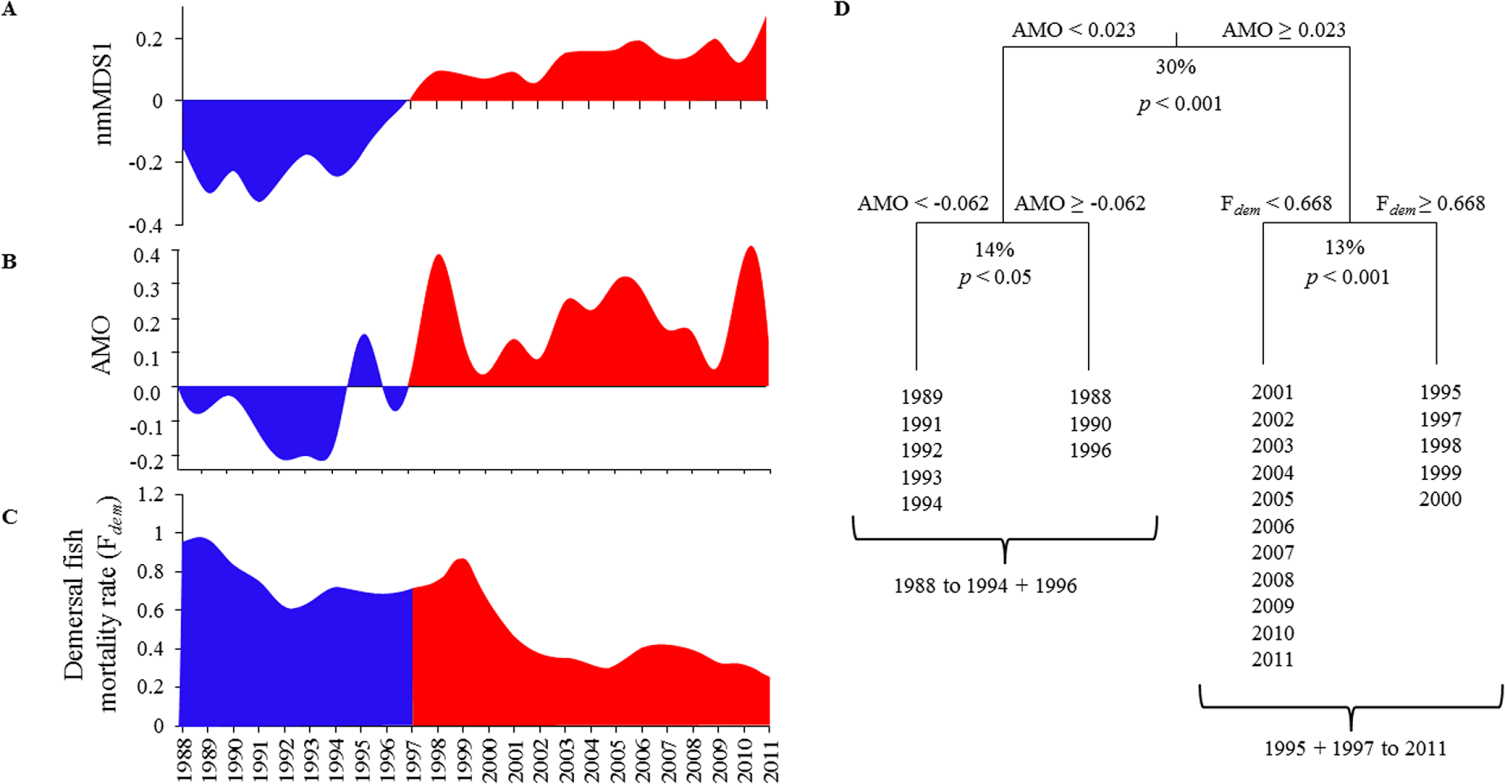
Relationship between the fish community dynamics and potential forcing factors. **A**. Evolution of community structure represented by values on the nmMDS first axis **B**. AMO time series **C**. F_*demersal*_ time series. **D**. MRT of the fish community structure time series on AMO and F_*demersal*_. The blue and red shaded areas correspond to the period before and after the regime shift, respectively.

A redundancy analysis followed by stepwise reduction allowed the identification of the most influential variables on fish community structure among the 9 tested factors (AMO, NAO, GNSW, SST, SSS, SSO2, F_*pelagic*_, F_*demersal*_, F_*benthic*_; see time series in S3 Fig). The most parsimonious model included AMO and demersal fishing mortality (F_*demersal*_) as explanatory variables, which accounted for 44.4% of temporal variations in community structure (Fig 3 and S2 Fig). Note that no sign of temporal (partial) autocorrelation was found in explained variables. Variation partitioning revealed almost equal contributions of the two factors to variability, AMO explaining 29.3% and F_*demersal*_ contributing 26.6%. However, the shift in fish community structure (summarized by values on the nmMDS first axis, Fig 3A) occurred concomitantly with a switch of the AMO from a cool to a warm phase between 1994 and 1998 (Fig 3B) whereas it was asynchronous with notable drops in F_*demersal*_ between 1989 and 1992 and between 1999 and 2005 (Fig 3C). A MRT on AMO and F_*demersal*_ identified that the community structure time series could be first subdivided into two groups of years based on AMO values, which accounted for 30% of variability (permutation test: p < 0.001; Fig 3D). These groups corresponded to the years before and after the regime shift, indicating that it was primarily linked to the AMO. F_*demersal*_ explained shorter-term variations in fish community structure, generating a second tree division 3–4 years after the shift (permutation test: *p* < 0.001; Fig 3D).

## Discussion

Our analyses revealed a rapid and intense change in the sampled structure of the EEC fish community in the mid-1990s that was synchronous with a change from a cold to a warm phase of a low-frequency natural climate cycle, namely the AMO, in the North Atlantic Ocean [18, 28]. This regime shift is characterized in the EEC by a decrease of small-bodied fishes. As other studies in the area recorded a geographical shift of small pelagic species following AMO cycles [28], we infer that the decrease we document is mainly caused by northward movements of small fish possibly as a response to warmer water temperature [11]. To our knowledge, this is the first study that explicitly demonstrates a regime shift of an entire fish community structure at the ecosystem scale in the Northeast Atlantic and that relates it to low-frequency natural climate oscillations. Intense fishing, on the other hand, revealed no direct influence on this response.

The rapid mid-1990s climatic change in the North Atlantic is associated to hydrographic regimes comparable to previous AMO switches [59]. Catches of small pelagic fish (i.e., anchovy, sardine, sardinella and herring) in the eastern North, Central Atlantic and Mediterranean Seas vary in synchrony with the warm and cool AMO phases due to changes in abundances and northward migrations particularly in the mid-1990s [28]. The period is similarly marked by an increase in abundance of anchovies and sardines in the North, Baltic and Wadden Seas [60,61]. In adjacent seas, Norwegian spring-spawning herrings showed important variations in abundance during the last century which have been mainly cued by temperature variations due to inflowing warm water mass movements to the north and east [62]. Such movements are mainly caused by an abrupt contraction of the subpolar gyre [28]. In the western English Channel, the AMO cycle is largely synchronous to the Russell Cycle [24,32] that characterizes profound changes in abundance and species distribution shifts in the area since the mid-19^th^ century [30]. Some authors [22,30] indicated benthic and pelagic species (i.e., sardines, bream, red mullet, macro/meso zooplankton and barnacles) shifts that occurred during the positive AMO-Russell cycle synchrony may have been driven by wider underlying forces, perhaps influencing circulation patterns and flow directions into the Channel aside from the more proximate effects of SST. Another comparable study [10] at a smaller scale (Plymouth Sound in the Western English Channel), showed a clear response of the demersal fish community to climatic fluctuations and especially to the increase in SST from 1990 to 2010.

Regime shifts were rarely related to low-frequency climate variations (such as the AMO), especially in the Northeast Atlantic where most studies insisted on the importance of high-frequency climate modes (e.g. the NAO; [6]). The AMO is an important indicator of long-term climate variability throughout the North Atlantic [26, 27] revealing multi-decadal variations in SST although other factors may be considered in the analysis of SST variability [22]. Contrary to the NAO that indicates several climatic components (precipitations, direction/intensity of wind, etc.), the AMO seems more relevant for explaining climate-induced regime shifts since sea temperature is considered as one of the major factors behind biotic shifts in phytoplankton [63,64], zooplankton [6] and fish [11]. However, NAO and AMO are partly linked through atmospheric-oceanic circulation coupling since SST depends and acts on atmospheric circulation [18,27,65]. Furthermore, beyond its role as a simple indicator of the North Atlantic SST, the AMO is progressively also considered as a ‘proxy for complex processes in the coupled atmosphere-ocean system of the North Atlantic’ manifested among others by large-scale changes in current direction and intensity throughout the North Atlantic and likely involves the NAO, the Atlantic Meridional Overturning Circulation (AMOC), the Mediterranean Overflow Water (MOW) and the subpolar gyre [28,59]. All such processes may induce biotic shifts, but ideally causal implication of the AMO can only be verified through particularly long-term survey datasets that cover at least one AMO cycle (60–80 years). In this study, the dataset considered is of moderate length (24 years) covering only a part of one AMO cycle, which may limit direct inference to the causal link of between this climate index and the regime shift in demersal community structure observed in the EEC. Caution must also be taken on assuming ecosystem-level responses based on long-term large-scale SST variation alone as other processes may be involved [22,23].

Despite our moderate-length time series compared to the AMO cycle period, we were able to distinguish a link between species’ abundance changes and their temperature preferendum supporting to some extent the hypothesis of a climate-induced effect. We observed that small-bodied highly fecund fish species supported by short plankton-based food chain were the most heavily impacted. This observation is consistent with the high sensitivity of such species to climatic fluctuations [61,66]. In addition to the possibility of change in some species’ migration phenology that could modify the community observed at survey time, several non-mutually exclusive processes may explain the climate-driven regime shift [67].

First, potential changes in fish distribution may have occurred as a response to the positive AMO phase as observed in several studies [61,68] and/or to climate-induced latitudinal shifts in food resources (e.g. zoo-planktonic communities; [6,11]). Fish movements may have been favored by northeastward inflowing water masses into the Channel in the mid-1990s from the Bay of Biscay which characterizes the beginning of a new warm phase (positive AMO index values) [30]. We observed opposite trends contrary to those presented by other authors in terms of small-sized pelagic fish abundance as a response to positive AMO index in the Western English Channel [28] and in the North and Baltic Seas [60,61] where anchovy and/or sardine increased in abundance. In the North Sea, other small-sized pelagic and demersal fish species such as mackerel, red gurnard, red mullet, tub gurnard, bluemouth, bib, John dory, poor cod have also shown sudden, almost exponential, increases in abundance since the mid-1990s [68,69]. These authors attributed this response to climatically driven expansion of fish migration range from the Channel to the North Sea. Such poleward movements of small-sized pelagic and demersal species during positive phases of the AMO seem to support our results and may explain their decline in the EEC. In contrast, we detected a decrease in EEC sprat abundance similar to observations in the North and Baltic Seas during positive AMO phase [60]. Spatial distribution shifts are known to be strongly correlated with variations in SST [70]. In the northeast Atlantic, several studies showed that Lusitanian fishes moved northwards into the English Channel, Celtic and North Seas over the last few decades [11,61,71,72]. This hypothesis could therefore be tested by investigating spatio-temporal changes in prey species as well as the potential northward shift of adults from the EEC to the North Sea.

Second, fish recruitment may also have been affected. Today, there exists an accumulation of evidence that points to the AMO as a regulator of fish biomass through bottom-up effects on recruitment dynamics [71–74]. We propose three different mechanisms that could explain potential impacts of the AMO on fish recruitment: (i) temperature variation affecting larval and/or juvenile mortality rates [73]; (ii) changes in spawning and/or productivity timing affecting the match-mismatch between larvae and their food resources and/or predators [74]; and (iii) modifications of hydrodynamic connectivity between habitats at successive life-stages affecting life-cycle closure, and especially larval dispersal patterns [73] that may have been influenced by the exceptional inflow of oceanic water in the mid-1990s.

Third, physiological responses of bioenergetic rates may have also directly or indirectly (through growth) modified the balance between fecundity, mortality and maturation rates and, thus, impacted population dynamics [67], and, as a result, community dynamics. A temperature increase may have improved fecundity and juvenile growth rate resulting in higher recruitments and abundance [23] of large-bodied demersal fish species in this case study. Additional analyses on population and community size spectra would allow investigating this physiological hypothesis in further details. Similarly, more detailed analyses based not only on species’ average, thermal preferences but on their entire thermal spectra would be helpful for better interpreting the role played by temperature in the observed regime shift.

Aside from environmental factors, community structure changes can be influenced by density-dependent interactions such as predation and competition, notably within the pelagic compartment of the ecosystem. Climate-induced changes in seasonal timing of plankton production during the positive AMO phase may have also contributed to altered fish community structure in the EEC. This may have affected the population dynamic response as recruitment declines due to a match-mismatch coupled with low local larval retention mechanisms [18,74]. This has consequences for plankton predators, including fish, whose life cycles are dependent on feeding on this seasonal production [18,59,67]. Long-term changes in relative abundance and community structure of offshore plankton in the English Channel have been linked to fluctuations in SST, NAO, subpolar gyre contraction [59,75] and advection [31]. Zooplankton composition in the western English Channel showed a shift from large to small-sized copepods with warm water affinities during the positive AMO phase [59,76]. In the North Sea, a climate-induced regime shift was observed in the late 80s [3] with a change in plankton communities [59,77]. The latter was attributed to a coincident increase in water flow and temperature of the European slope current that brought unusually warm and nutrient-rich water into the ecosystem [78], which was reflected in the positive AMO in our study. Observed ecological responses included the decline of European cod due to climate warming and climate-induced changes in plankton production coupled with intense exploitation [18,42].

Despite its significant effect on variability in fish community composition, exploitation did not seem to be the proximal cause of the regime shift observed in the area because of a clear asynchrony between fishing mortality variations and this shift. Additionally, the shift was characterized by a decrease in small-bodied low trophic level fish and an increase in large-sized species at higher trophic levels, which is opposite to classical shifts in size-structure observed in exploited fish communities, i.e. the upper-end truncation of the size and/or trophic spectrum [34]. Furthermore, fishing mortality has declined in the northeast Atlantic since 1993 which may support the hypothesis that climate, and not overfishing, triggered the structural shift in fish community [72]. We, however, do not exclude that historical fishing primed the system for the observed regime shift. The long history of exploitations in the EEC may indeed have facilitated the regime shift by favoring the dominance of small-bodied low trophic level species that are known to have the fastest demographic responses to environmental variations, especially temperature [11], thereby rendering the whole fish community more sensitive to climatic variations [33]. Beyond a potential implication in the regime shift, the decrease in fishing pressure on large demersal fish 4 years after the shift may have contributed in maintaining the increased abundance of this group.

The important regime shift detected may have consequences on the EEC ecosystem structure and functioning across multiple scales of biological organization. First, the increase of some piscivorous large demersal species may impose supplementary predation pressure on small forage species leading to cascading ecological effects on lower (plankton) or higher trophic level compartments (larger fishes, seabirds or marine mammals) [66]. Second, abundance decline of small forage species compared to larger demersal species could have occurred due to size-dependent metabolism [79]. Third, increased abundance of large predatory fish species and enhanced diversity might contribute to ecosystem resilience and partly counteract the destabilizing effect of exploitation on fish community dynamics [80]. However, despite their increase during the shift, large fish species abundance remains at relatively low levels and may still be vulnerable to unsustainable fishing pressure or unfavorable environmental conditions [74]. Ultimately, such an alteration in the structure of fish communities is very likely to impact the socio-economic nature and value of commercial fisheries [11].

To conclude, this paper illustrates that low-frequency (multidecadal) climate variations may be related to regime shifts affecting fish communities whereas we found no relationship with high-frequency (intradecadal) climate variability. The regime shift detected in this study was clearly related to natural multidecadal variation in the North Atlantic SST and water flows as described by the AMO but was probably facilitated by heavy historic exploitation that impacted a more climate-sensitive fish community. This link with the AMO is in agreement with the observation by others that a series of complex climatic processes that occurred in the North Atlantic starting in the 1960s and then culminating in the 1990s had pervasive ecosystem and ecological repercussions [28]. We, however, cannot exclude that the substantial fish community reorganization observed was a consequence of gradual trends in one or several drivers [55] and not of a rapid climate-change revealed by the AMO. Nonetheless, the observed fish community restructuration also supports the fact that local ecological variability in the EEC is indicative of large-scale climatic processes [22,30] and adds to the evidence of the Russell Cycle. Local conditions are likely to degrade further for Boreal species due to global warming and attention should be paid to interactions with both natural climate variability and fishing when aiming at sustainable exploitation and biodiversity conservation. Furthermore, describing the dynamics and shifts of plankton-fish relationship using long time-series data would be useful to better understand the mechanisms associated to the observed regime shift [59,75,81,82]. Last but not least, the observed taxonomic regime shift in the EEC fish community may also correspond to a functional shift, the characteristics of which should be determined and the consequences for ecosystem functioning and exploitation sustainability investigated.

## Supporting Information

S1 Fig Channel Ground Fish Survey (CGFS) sampling scheme. Locations of sampling stations (crosses) are presented for the CGFS time series from 1988 to 2011 with the spatial stratification grid superimposed. Each rectangle measures 15’×15’ and is sampled at least once per year, when the nature of the bottom allows it.
(DOCX)

S2 Fig Relationship between temporal variability in community structure, environmental conditions and fishing pressure. A RDA biplot on the two first axes presents temporal variability in community structure (year labels) and its relationship with the two selected variables (arrows), AMO and F_demersal_, after stepwise model reduction. The shift in community structure can clearly be seen along axis 1.
(DOCX)

S3 Fig Dynamics of fish community structure, environmental conditions and fishing pressure. All data are centered and scaled except for panel A. A. Projection of the species abundance matrix on the first axis of the non-metric multidimensional scaling as illustration of the dynamics of the community structure. B. Atlantic Multidecadal Oscillation. C. North Atlantic Oscillation. D. Gulf Stream North Wall. E. Mean annual temperature. F. Mean annual salinity. G. Mean annual dissolved oxygen concentration. H. Pelagic fishing mortality rate. I. Demersal fishing mortality rate. J. Benthic fishing mortality rate. Only annual mean values of physico-chemical parameters are shown in order to ease readability. The blue and red shaded areas correspond to the period before and after the regime shift, respectively.
(DOCX)

S1 Table Summary of environmental and fishing pressure data. For each parameter considered, its name, abbreviation, geographical scale, the source and type of data used, and the URL where these data are available are given.
(DOCX)

S2 Table List of the 55 taxa used in this study. For each taxon, the scientific and common names, the position in the water column, the mean total body length and the thermal preferendum are given.—: taxa for which no individual-level total length data are available.
(DOCX)

S3 Table Results of the Piecewise linear Regression applied to taxonomic richness, evenness and diversity index values. (DOCX)

S4 Table Results of the Piecewise linear Regression applied to mean body length in fish community. (DOCX)
