# Supplementary material for: Regime Shift in an Exploited Fish Community Related to Natural Climate Oscillations
Source: PLoS One. 2015 Jul 1;10(7):e0129883. doi: 10.1371/journal.pone.0129883 (PMC4488883; doi:10.1371/journal.pone.0129883)
Supplement: S1 Fig — Locations of sampling stations (crosses) are presented for the CGFS time series from 1988 to 2011 with the spatial stratification grid superimposed. Each rectangle measures 15’×15’ and is sampled at least once per year, when the nature of the bottom allows it. (DOCX) [file pone.0129883.s001.docx]

S1 Fig. Channel Ground Fish Survey (CGFS) sampling scheme. Locations of sampling stations (crosses) are presented for the CGFS time series from 1988 to 2011 with the spatial stratification grid superimposed. Each rectangle measures 15’×15’ and is sampled at least once per year, when the nature of the bottom allows it.
