# Supplementary material for: Regime Shift in an Exploited Fish Community Related to Natural Climate Oscillations
Source: PLoS One. 2015 Jul 1;10(7):e0129883. doi: 10.1371/journal.pone.0129883 (PMC4488883; doi:10.1371/journal.pone.0129883)
Supplement: S2 Fig — A RDA biplot on the two first axes presents temporal variability in community structure (year labels) and its relationship with the two selected variables (arrows), AMO and Fdemersal, after stepwise model reduction. The shift in community structure can clearly be seen along axis 1. (DOCX) [file pone.0129883.s002.docx]

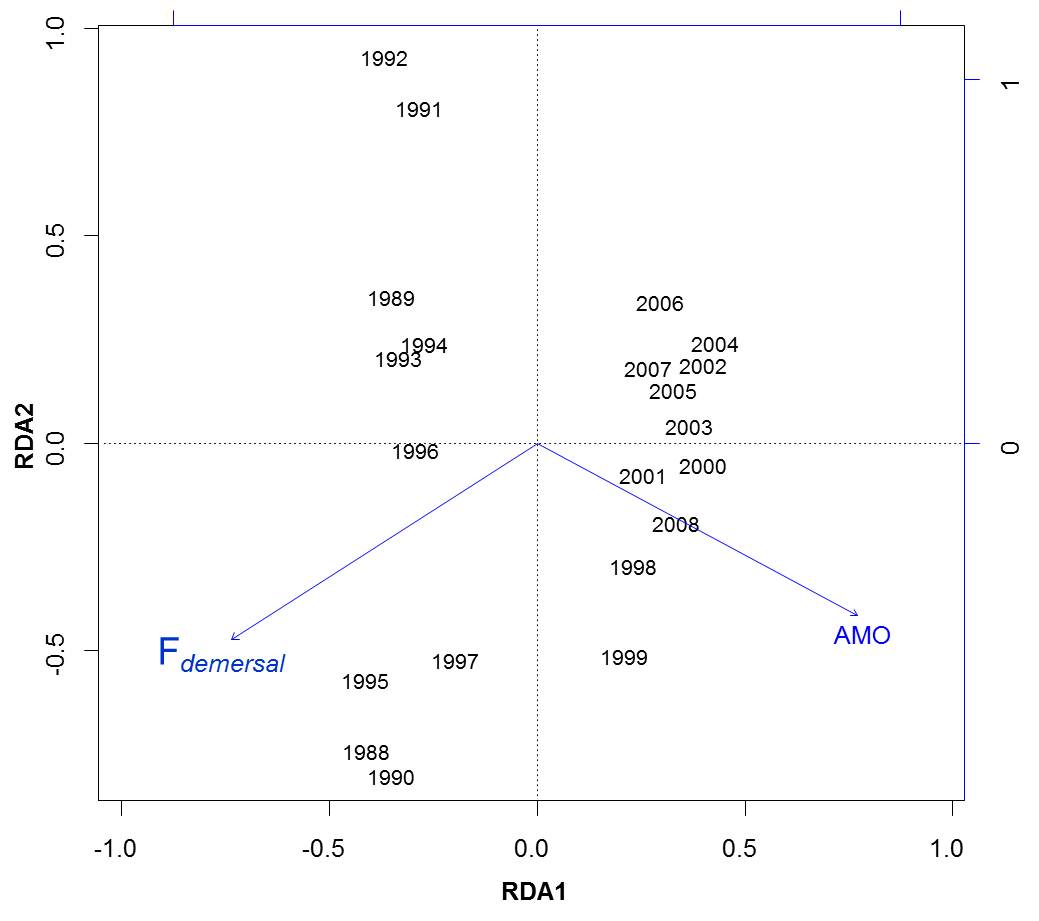


S2 Fig. Relationship between temporal variability in community structure, environmental conditions and fishing pressure. A RDA biplot on the two first axes presents temporal variability in community structure (year labels) and its relationship with the two selected variables (arrows), AMO and F_demersal_, after stepwise model reduction. The shift in community structure can clearly be seen along axis 1.
