# Supplementary material for: Regime Shift in an Exploited Fish Community Related to Natural Climate Oscillations
Source: PLoS One. 2015 Jul 1;10(7):e0129883. doi: 10.1371/journal.pone.0129883 (PMC4488883; doi:10.1371/journal.pone.0129883)
Supplement: S3 Fig — All data are centered and scaled except for panel A. A. Projection of the species abundance matrix on the first axis of the non-metric multidimensional scaling as illustration of the dynamics of the community structure. B. Atlantic Multidecadal Oscillation. C. North Atlantic Oscillation. D. Gulf Stream North Wall. E. Mean annual temperature. F. Mean annual salinity. G. Mean annual dissolved oxygen concentration. H. Pelagic fishing mortality rate. I. Demersal fishing mortality rate. J. Benthic fishing mortality rate. Only annual mean values of physico-chemical parameters are shown in order to ease readability. The blue and red shaded areas correspond to the period before and after the regime shift, respectively. (DOCX) [file pone.0129883.s003.docx]

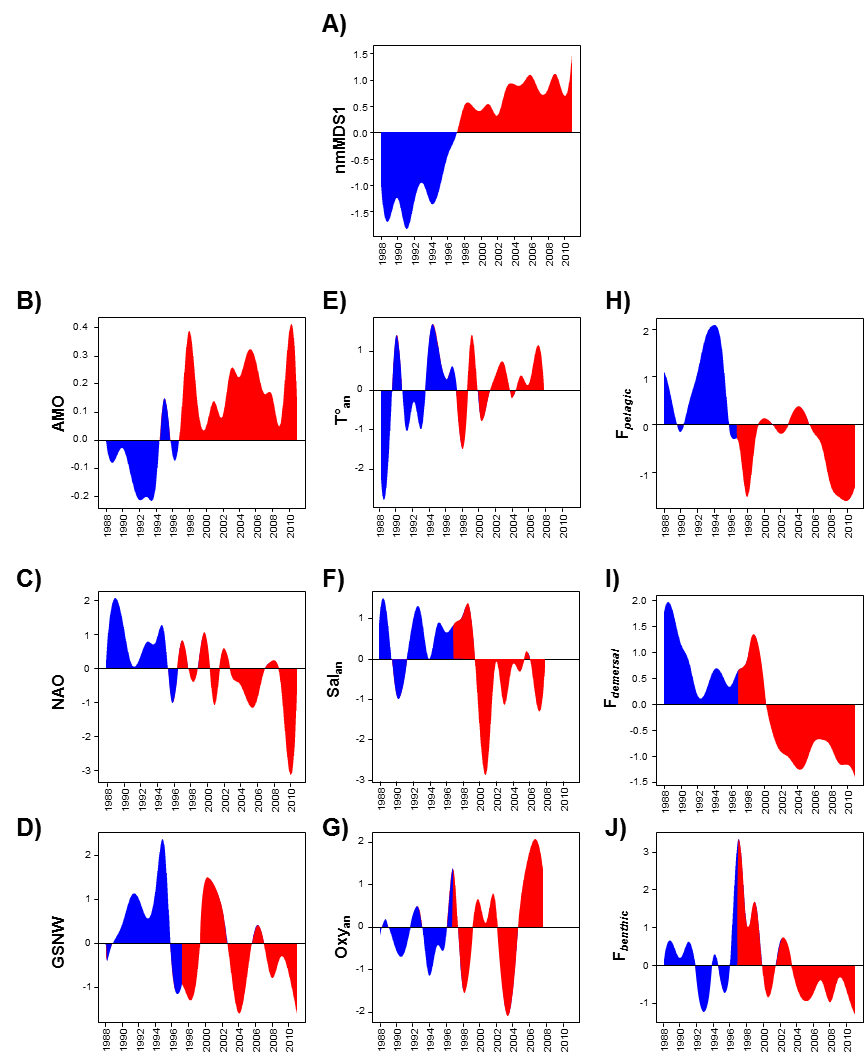


S3 Fig. Dynamics of fish community structure, environmental conditions and fishing pressure. All data are centered and scaled except for panel A. A. Projection of the species abundance matrix on the first axis of the non-metric multidimensional scaling as illustration of the dynamics of the community structure. B. Atlantic Multidecadal Oscillation. C. North Atlantic Oscillation. D. Gulf Stream North Wall. E. Mean annual temperature. F. Mean annual salinity. G. Mean annual dissolved oxygen concentration. H. Pelagic fishing mortality rate. I. Demersal fishing mortality rate. J. Benthic fishing mortality rate. Only annual mean values of physico-chemical parameters are shown in order to ease readability. The blue and red shaded areas correspond to the period before and after the regime shift, respectively.
