# Supplementary material for: Regime Shift in an Exploited Fish Community Related to Natural Climate Oscillations
Source: PLoS One. 2015 Jul 1;10(7):e0129883. doi: 10.1371/journal.pone.0129883 (PMC4488883; doi:10.1371/journal.pone.0129883)
Supplement: S1 Table — For each parameter considered, its name, abbreviation, geographical scale, the source and type of data used, and the URL where these data are available are given. (DOCX) [file pone.0129883.s004.docx]

S1 Table. Summary of environmental and fishing pressure data. For each parameter considered, its name, abbreviation, geographical scale, the source and type of data used, and the URL where these data are available are given.

|  |  |  |  |  |  |  |  |  |  |  |  |  |  |  |
| --- | --- | --- | --- | --- | --- | --- | --- | --- | --- | --- | --- | --- | --- | --- |
| parameter name | |  | abbreviation |  | geographical scale | |  | source of data | |  | type of data |  | URL | |
| mean annual temperature | |  | SST_an_ |  | Local (Eastern English Channel) | |  | Institute of Marine Research | |  | output of the NORWECOM model |  | http://www.imr.no/~morten/wgoofe/ | |
| average temperature in October | |  | SST_oct_ |  |  |  |  |  |  |  |  |  |  |  |
| minimal temperature of the year | |  | SST_min_ |  |  |  |  |  |  |  |  |  |  |  |
| maximal temperature of the year | |  | SST_max_ |  |  |  |  |  |  |  |  |  |  |  |
| mean annual salinity | |  | SSS_an_ |  |  |  |  |  |  |  |  |  |  |  |
| average salinity in October | |  | SSS_oct_ |  |  |  |  |  |  |  |  |  |  |  |
| minimal salinity of the year | |  | SSS_min_ |  |  |  |  |  |  |  |  |  |  |  |
| maximal salinity of the year | |  | SSS_max_ |  |  |  |  |  |  |  |  |  |  |  |
| annual mean of dissolved oxygen concentration | |  | SSO2_an_ |  |  |  |  |  |  |  |  |  |  |  |
| monthly average of dissolved oxygen concentration in October | |  | SSO2_oct_ |  |  |  |  |  |  |  |  |  |  |  |
| minimal dissolved oxygen concentration of the year | |  | SSO2_min_ |  |  |  |  |  |  |  |  |  |  |  |
| maximal dissolved oxygen concentration of the year | |  | SSO2_max_ |  |  |  |  |  |  |  |  |  |  |  |
| Atlantic Multidecadal Oscillation | |  | AMO |  | Global (North Atlantic ocean) | |  | National Oceanic and Atmospheric Administration | |  | index |  | http://www.esrl.noaa.gov/psd/data/timeseries/AMO/ | |
| North Atlantic Oscillation | |  | NAO |  |  |  |  |  |  |  |  |  | http://www.cpc.ncep.noaa.gov/products/precip/CWlink/pna/nao_index.html | |
| Gulf Stream North Wall | |  | GSNW |  |  |  |  |  |  |  |  |  | http://www.pml.ac.uk/gulfstream | |
| Pelagic fishing mortality rate | |  | F*_pelagic_* |  | Regional | |  | International Council for the Exploitation of the Sea | |  | output of stock assessment models |  | http://www.ices.dk/fish/CATChSTATISTICS.asp | |
| Demersal fishing mortality rate | |  | F*_demersal_* |  |  |  |  |  |  |  |  |  |  |  |
| Benthic fishing mortality rate | |  | F*_benthic_* |  |  |  |  |  |  |  |  |  |  |  |
|  |  |  |  |  |  |  |  |  |  |  |  |  |  |  |
