# Supplementary material for: Regime Shift in an Exploited Fish Community Related to Natural Climate Oscillations
Source: PLoS One. 2015 Jul 1;10(7):e0129883. doi: 10.1371/journal.pone.0129883 (PMC4488883; doi:10.1371/journal.pone.0129883)
Supplement: S2 Table — For each taxon, the scientific and common names, the position in the water column, the mean total body length and the thermal preferendum are given.—: taxa for which no individual-level total length data are available. (DOCX) [file pone.0129883.s005.docx]

**S2 Table.** List of the 55 taxa used in this study. For each taxon, the scientific and common names, the position in the water column, the mean total body length and the thermal preferendum are given. - : taxa for which no individual-level total length data are available.

|  |  |  |  |  |  |  |  |  |  |  |  |  |  |  |  |  |  |  |
| --- | --- | --- | --- | --- | --- | --- | --- | --- | --- | --- | --- | --- | --- | --- | --- | --- | --- | --- |
| Species name |  | Common name |  | Position in water column |  | Mean body length (cm) |  | Thermal preferendum (°C) |  | Species |  | Common name |  | Position in water column |  | Mean body length (cm) |  | Thermal preferendum (°C) |
| *Agonus cataphractus* |  | hooknose |  | benthic |  | - |  | 11.3 |  | *Pleuronectes platessa* |  | plaice |  | benthic |  | 27.9 |  | 10.8 |
| *Alosa spp.* |  | Allis shad |  | pelagic |  | 32.6 |  | 11.1 |  | *Pollachius pollachius* |  | pollack |  | demersal |  | 37.2 |  | 11.6 |
| *Anguilla anguilla* |  | common eel |  | pelagic |  | 58.1 |  | 10.7 |  | *Psetta maxima* |  | turbot |  | benthic |  | 31.6 |  | 10.2 |
| *Aspitrigla cuculus* |  | red gurnard |  | benthic |  | 27.5 |  | 12.5 |  | *Raja clavata* |  | thornback ray |  | benthic |  | 53.9 |  | 13.8 |
| *Atherinidae* |  | sand smelts |  | pelagic |  | 12.0 |  | 14.5 |  | *Raja brachyura* |  | blonde ray |  | benthic |  | 51.1 |  | 15.0 |
| *Buglossidium spp.* |  | solenette |  | benthic |  | - |  | 12.8 |  | *Raja montagui* |  | spotted ray |  | benthic |  | 47.4 |  | 12.5 |
| *Callionymus spp.* |  | Dragonet |  | benthic |  | 17.6 |  | 11.6 |  | *Raja naevus* |  | cuckoo ray |  | benthic |  | 48.5 |  | 14.0 |
| *Centrolabrus spp.* |  | rock cook |  | demersal |  | 16.7 |  | 11.9 |  | *Raja undulata* |  | undulate ray |  | benthic |  | 59.2 |  | 15.3 |
| *Clupea harengus* |  | herring |  | pelagic |  | 14.7 |  | 8.5 |  | *Sardina pilchardus* |  | sardine |  | pelagic |  | 16.4 |  | 18.8 |
| *Dasyatis pastinaca* |  | common stingray |  | benthic |  | 67.1 |  | 19.3 |  | *Scomber scombrus* |  | mackerel |  | pelagic |  | 31.1 |  | 10.4 |
| *Dicentrarchus labrax* |  | seabass |  | pelagic |  | 38.3 |  | 12.8 |  | *Scophthalmus rhombus* |  | brill |  | benthic |  | 29.4 |  | 11.5 |
| *Engraulis encrasicolus* |  | anchovy |  | pelagic |  | 10.2 |  | 25.5 |  | *Scyliorhinus canicula* |  | small-spotted dogfish |  | demersal |  | 54.5 |  | 11.3 |
| *Eutrigla gurnardus* |  | grey gurnard |  | benthic |  | 22.8 |  | 11.3 |  | *Scyliorhinus stellaris* |  | nursehound |  | demersal |  | 59.6 |  | 23.4 |
| *Gadus morhua* |  | cod |  | demersal |  | 49.4 |  | 7.9 |  | *Sepia spp.* |  | cuttlefish |  | benthic |  | - |  | 14.6 |
| *Galeorhinus galeus* |  | tope |  | demersal |  | 85.5 |  | 17.3 |  | *Solea solea* |  | Sole |  | benthic |  | 22.4 |  | 11.2 |
| *Hyperoplus spp.* |  | greater sandeel |  | demersal |  | 23.4 |  | 12.3 |  | *Spondyliosoma cantharus* |  | black seabream |  | demersal |  | 19.2 |  | 16.2 |
| *Limanda limanda* |  | dab |  | benthic |  | 21.3 |  | 11.1 |  | *Sprattus sprattus* |  | sprat |  | pelagic |  | 9.8 |  | 9.9 |
| *Liza aurata* |  | golden grey mullet |  | pelagic |  | 44.0 |  | 17.5 |  | *Squalus acanthias* |  | picked dogfish |  | demersal |  | 68.8 |  | 11.5 |
| *Loligo spp.* |  | european squid |  | demersal |  | - |  | 17.5 |  | *Trachinus draco* |  | greater weever |  | benthic |  | 30.2 |  | 13.9 |
| *Lophius piscatorius* |  | angler fish |  | benthic |  | 60.3 |  | 20.9 |  | *Trachinus vipera* |  | lesser weever |  | benthic |  | 11.0 |  | 13.4 |
| *Merlangius merlangus* |  | Whiting |  | demersal |  | 26.8 |  | 10.6 |  | *Trachurus trachurus* |  | horse mackerel |  | pelagic |  | 20.4 |  | 17.7 |
| *Microchirus variegatus* |  | thickback sole |  | benthic |  | 14.6 |  | 15.7 |  | *Trigla lucerna* |  | tub gurnard |  | benthic |  | 28.7 |  | 13.8 |
| *Microstomus kitt* |  | lemon sole |  | benthic |  | 24.4 |  | 11.0 |  | *Trigla lyra* |  | piper gurnard |  | benthic |  | 32.2 |  | 26.8 |
| *Molva molva* |  | ling |  | demersal |  | 79.4 |  | 10.9 |  | *Trigloporus lastoviza* |  | rock gurnard |  | benthic |  | - |  | 25.3 |
| *Mullus surmuletus* |  | red mullet |  | benthic |  | 19.6 |  | 16.2 |  | *Trisopterus luscus* |  | pouting |  | demersal |  | 18.5 |  | 11.5 |
| *Mustelus asterias* |  | starry smoothhound |  | demersal |  | 67.0 |  | 12.9 |  | *Trisopterus minutus* |  | poor cod |  | demersal |  | 12.0 |  | 11.8 |
| *Mustelus mustelus* |  | smoothhound |  | demersal |  | 71.9 |  | 22.4 |  | *Zeus faber* |  | John dory |  | demersal |  | 25.6 |  | 18.9 |
| *Platichthys flesus* |  | flounder |  | benthic |  | 28.5 |  | 9.7 |  |  |  |  |  |  |  |  |  |  |
|  |  |  |  |  |  |  |  |  |  |  |  |  |  |  |  |  |  |  |
|  |  |  |  |  |  |  |  |  |  |  |  |  |  |  |  |  |  |  |
