# Supplementary material for: Regime Shift in an Exploited Fish Community Related to Natural Climate Oscillations
Source: PLoS One. 2015 Jul 1;10(7):e0129883. doi: 10.1371/journal.pone.0129883 (PMC4488883; doi:10.1371/journal.pone.0129883)
Supplement: S3 Table — (DOCX) [file pone.0129883.s006.docx]

S3 Table. Results of the Piecewise linear Regression applied to taxonomic richness, evenness and diversity index values.

|  |  |  |  |  |  |  |  |  |  |  |
| --- | --- | --- | --- | --- | --- | --- | --- | --- | --- | --- |
| Community parameter ~ *a**year + b | | | | | | | | | | |
|  |  |  |  | year < breakpoint | | |  | year > breakpoint | | |
| Community parameter |  | Parameters |  | *a* |  | *b* |  | *a* |  | *b* |
| Richness (breakpoint = 1991; F^4^_19_ = 0.478; *p* = 0.751) |  | Estimate |  | 0.018 |  | 12.854 |  | 0.0439 |  | -38.681 |
|  |  | Std. Error |  | 0.138 |  | 275.17 |  | 0.057 |  | 115.022 |
|  |  | t value |  | 0.132 |  | 0.047 |  | 0.766 |  | -0.336 |
|  |  | Pr(>│t│) |  | 0.898 |  | 0.964 |  | 0.458 |  | 0.742 |
| Evenness (breakpoint = 1998; F^4^_19_ = 106.1; *p* < 0.001) |  | Estimate |  | 0.012 |  | -23.885 |  | 0.002 |  | -5.173 |
|  |  | Std. Error |  | 0.005 |  | 10.286 |  | 0.001 |  | 2.554 |
|  |  | t value |  | 2.322 |  | -2.322 |  | 2.301 |  | -2.026 |
|  |  | Pr(>│t│) |  | 0.045 (*) |  | 0.048 (*) |  | 0.0401 (*) |  | 0.065 |
| Diversity (breakpoint = 1998; F^4^_19_ = 103; *p* < 0.001) |  | Estimate |  | 0.047 |  | -92.452 |  | 0.012 |  | -21.437 |
|  |  | Std. Error |  | 0.021 |  | 41.275 |  | 0.005 |  | 10.481 |
|  |  | t value |  | 2.279 |  | -2.24 |  | 2.308 |  | -2.045 |
|  |  | Pr(>│t│) |  | 0.052 |  | 0.055 |  | 0.039 (*) |  | 0.063 |
|  |  |  |  |  |  |  |  |  |  |  |
