# Supplementary material for: Regime Shift in an Exploited Fish Community Related to Natural Climate Oscillations
Source: PLoS One. 2015 Jul 1;10(7):e0129883. doi: 10.1371/journal.pone.0129883 (PMC4488883; doi:10.1371/journal.pone.0129883)
Supplement: S4 Table — (DOCX) [file pone.0129883.s007.docx]

S4 Table. Results of the Piecewise linear Regression applied to mean body length in fish community.

|  |  |  |  |  |  |  |  |  |  |  |
| --- | --- | --- | --- | --- | --- | --- | --- | --- | --- | --- |
| mean body length in fish community ~ *a**year + b | | | | | | | | | | |
|  |  |  |  | year < breakpoint | | |  | year > breakpoint | | |
|  |  | Parameters |  | *a* |  | *b* |  | *a* |  | *b* |
| (breakpoint = 1998; F^3^_20_ = 34.67; *p* < 0.001) |  | Estimate |  | -0.005 |  | 27.32 |  | 0.147 |  | -273.04 |
|  |  | Std. Error |  | 0.148 |  | 295.42 |  | 0.047 |  | 95.38 |
|  |  | t value |  | -0.034 |  | 0.092 |  | 3.087 |  | -2.863 |
|  |  | Pr(>│t│) |  | 0.973 |  | 0.929 |  | 0.009 |  | 0.014 |
|  |  |  |  |  |  |  |  |  |  |  |
